# Predictors of mortality among HIV infected patients taking antiretroviral treatment in Ethiopia: a retrospective cohort study

**DOI:** 10.1186/1742-6405-9-15

**Published:** 2012-05-18

**Authors:** Sibhatu Biadgilign, Ayalu A Reda, Tesfaye Digaffe

**Affiliations:** 1 Department of Epidemiology and Biostatistics, College of Medical and Health Science, Jimma University, Jimma, Ethiopia; 2 Department of Public Health, College of Health Sciences, Haramaya University, P. O. Box 235, Harar, Ethiopia; 3 Department of Medical Laboratory Science, College of Health Sciences, Harar, Ethiopia

## Abstract

**Background:**

Studies indicate that there is high early mortality among patients starting antiretroviral treatment in sub-Saharan Africa. However, there is paucity of evidence on long term survival of patients on anti-retroviral treatment in the region. The objective of this study is to examine mortality and its predictors among a cohort of HIV infected patients on anti-retroviral treatment retrospectively followed for five years.

**Methods:**

A retrospective cohort study was conducted among HIV infected patients on ART in eastern Ethiopia. Cox regression and Kaplan-Meier analyses were performed to investigate factors that influence time to death and survival over time.

**Result:**

A total of 1540 study participants were included in the study. From the registered patients in the cohort, the outcome of patients as active, deceased, lost to follow up and transfer out was 1005 (67.2%), 86 (5.9%), 210 (14.0%) and 192 (12.8%) respectively. The overall mortality rate provides an incidence density of 2.03 deaths per 100 person years (95% CI 1.64 - 2.50). Out of a total of 86 deaths over 60 month period; 63 (73.3%) died during the first 12 months, 10 (11.6%) during the second year, and 10 (11.6%) in the third year of follow up. In multivariate analysis, the independent predictors for mortality were loss of more 10% weight loss, bedridden functional status at baseline, ≤ 200 CD4 cell count/ml, and advanced WHO stage patients.

**Conclusion:**

A lower level of mortality was detected among the cohort of patients on antiretroviral treatment in eastern Ethiopia. Previous history of weight loss, bedridden functional status at baseline, low CD4 cell count and advanced WHO status patients had a higher risk of death. Early initiation of ART, provision of nutritional support and strengthening of the food by prescription initiative, and counseling of patients for early presentation to treatment is recommended.

## Introduction

Development of highly active antiretroviral treatment (ART) in the mid-1990s revolutionized the care of HIV-infected patients and led to marked reductions in HIV-associated morbidity and mortality in many industrialized countries [1,2]. ART has clearly shown to be effective in reducing mortality among those who remain in treatment and adhere to therapy [2-4]. In recent years in developing countries with a high burden of AIDS, ART has become more widely available. According to estimation by the World Health Organization (WHO), about 6 650 000 patients were receiving ART in low- and middle-income countries by the end of 2010 [5], this is a huge improvement from the levels in 2003 [6,7]. Two sub-Saharan African countries, Botswana and Rwanda, have achieved universal access target (treatment coverage of 80% or more of patients in need) at the end of 2009 [7], while countries such as Ethiopia, Zambia, Namibia and Senegal are moving closer to the same target having covered 50-80% of patients in need of treatment [7]. Due to this morbidity and mortality among HIV-infected persons have dramatically decreased [8,9].

The primary goals of antiretroviral therapy are preventing HIV-related morbidity and mortality, and improving quality of life by restoring immunologic function through suppression of viral load [10]. Many studies have reported high early mortality among patients starting antiretroviral treatment in sub-Saharan Africa [4]. One of the reasons cited for this is that despite better availability of ART, people are still diagnosed late and thus start ART at later stages of the HIV disease [11].

In Ethiopia, there were more than 222,000 patients on antiretroviral treatment at the end of 2010 [6]. ART has improved survival of patients with HIV/AIDS and improved the quality of life of patients in the country [12]. In Ethiopia, there are no studies reporting the long term survival of patients on antiretroviral treatment. Such studies could provide valuable information to evaluate the ART program in the country. The objective of this study is to examine mortality and its predictors among a cohort of HIV infected patients on antiretroviral treatment retrospectively followed for five years.

## Methods

### Study area and period

The study was conducted in Hiwot Fana, Jugal and Dil Chora hospitals located in eastern Ethiopia. Data was collected from September to November 2010.

### Study design

A retrospective cohort study was conducted among HIV patients on ART. A random sample of 1537 patients that started treatment between September 11, 2005 and September 10, 2008 were included in the study and retrospectively followed up for an additional two years until September 10, 2010. The patients’ identification numbers were used to generate the necessary sample from the records of the hospitals for extracting data.

Socio-demographic characteristics, baseline and follow up clinical and laboratory measurement information, and treatment outcomes were abstracted from patients' cards. The primary outcome measure was patient survival, while secondary outcome measures included CD4 count and body weight.

### Data collection and quality control

A standard questionnaire was used for recording information extracted from patients’ cards. This form is developed using the standardized ART entry and follow up form employed by the ART clinic. The CD4 count laboratory results recorded before starting ART were used as a base line values. If there is no pre-treatment laboratory test, however, results obtained within one month of ART initiation were considered as baseline values. Four experienced ART nurses who were trained on comprehensive HIV care and involved in patient follow ups collected the data. Data collection was supervised by the researchers. All completed data collection forms were examined for clarity and consistency. The data were entered and cleaned by trained data clerks and the investigators before analysis.

Death was ascertained by reviewing cards and death certificates. Patients who died from unrelated diseases or accident were considered as censored, as well as those alive at the end of the follow up period.

### Data analysis

Descriptive statistics such as median, mean, SDs and tables were be used to investigate the characteristics of the cohort. Person years of follow up were calculated by assessing the date of enrollment for ART and death or censoring. Survival analysis and the Kaplan-Meier test were used to investigate factors that influence time to death. Hazard ratios (HR), as well as 95% confidence intervals were used as effect measures. A p-value of 0.05 was used. Descriptive statistics and Cox regression were conducted using SPSS® version 16. Proportionality of hazards test on Schoenfeld residuals and graph were conducted using STATA® version 10.

### Ethical consideration

Ethical clearance was obtained from the Institutional Research Ethics Review Committee (IRERC) of Haramaya University. All information collected from patients cards were kept strictly confidential and names were not included in the abstracted data.

## Results

### Characteristics of patients

A total of 1540 study participants were included in the study. The sample comprised 963 (62.5%) females and 574 (37.3%) males respectively. The median and inter-quartile range (IQR) age was 32 and 28–40. The majority of participants 1074 (69.9%) were Orthodox Christians and 305 (19.8) were Muslim. Clinically, 890 (58.2%) and 348 (22.7%) patients were on the stage III and II. The median (IQR) baseline CD4 count was 135 (76.0–198.3) per milliliter (Table 1).

**Table 1:** Baseline characteristics of HIV infected patients initiating antiretroviral therapy

	All patients, ^¥^N (%)	Patients with CD4 count≤200 (n = 1149)	Patients with CD4 count>200 (n = 361)
Sex			
Male	574 (37.3)	431 (37.6)	131 (36.3)
Female	963 (62.5)	715 (62.4)	230 (63.7)
Age (Median, IQR)	32 (28–40)	32 (28.0–40.0)	35 (28.0–40.0)
Religion			
Muslim	305 (19.8)	220 (19.1)	76 (21.1)
Orthodox	1074 (69.9)	812 (70.7)	241 (66.8)
Protestant	144 (9.4)	103 (9.0)	41 (11.4)
Others	14 (0.9)	14 (1.2)	3 (0.8)
Education			
No education	261 (17.0)	185 (16.1)	67 (18.6)
Primary	681 (44.3)	490 (42.7)	178 (49.3)
Secondary	500 (32.5)	391 (34.1)	102 (28.3)
Tertiary	96 (6.2)	81 (7.1)	14 (3.9)
Marital status			
Never Married	287 (18.6)	217 (18.9)	65 (18.0)
Married	606 (39.4)	475 (41.4)	124 (34.3)
Separated	288 (18.7)	206 (17.9)	74 (20.5)
Divorced	116 (7.5)	89 (7.8)	22 (6.1)
Widowed	242 (15.7)	161 (14.0)	76 (21.1)
Occupation			
Merchant	75 (7.4)	60 (7.7)	15 (6.7)
Gov. Employee	157 (15.5)	122 (15.6)	33 (14.8)
Non-Gov. Employee	41 (4.1)	34 (4.4)	7 (3.1)
Day Laborer	174 (17.2)	130 (16.7)	44 (19.7)
Job-less	402 (39.7)	303 (38.8)	94 (42.2)
Other	163 (16.2)	131 (16.8)	30 (13.5)
Past co-trimoxazole treatment			
Yes	758 (49.2)	576 (50.1)	171 (47.4)
No	782 (50.8)	573 (49.9)	190 (52.6)
WHO stage at baseline			
Stage I	92 (6.0)	76 (6.7)	15 (4.2)
Stage II	348 (22.7)	282 (24.7)	63 (17.5)
Stage III	890 (58.2)	633 (55.5)	239 (66.6)
Stage IV	200 (13.1)	150 (13.1)	42 (11.7)
Baseline CD4 count, median (IQR)	135 (76.0–198.3)	107 (64.0–154.0)	253 (223.3–304.0)
Weight at baseline in kgs, median (IQR)	50.0 (44.0–56.0)	50 (44.0–56.0)	50 (45.0–57.0)

^¥^Number and percentages unless indicated otherwise. IQR, inter-quartile rage.

### Follow up and survival patterns of the cohort

**Figure 1:**
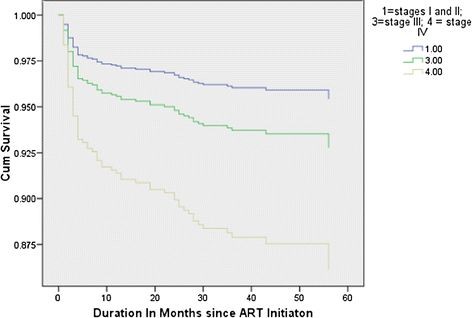
Survival functions stratified according to WHO clinical staging in HIV infected patients in a cohort of patients on antiretroviral treatment in eastern Ethiopia (y-axis truncated to improve visibility).

From the registered patients in this cohort analysis, the outcome of patients as active, deceased, lost to follow up and transfer out were 1005 (67.2%), 86 (5.9%), 210 (14.0%) and 192 (12.8%) respectively. There were 86 deaths in 4234.8 person years of retrospective follow up Figure 1 displays the survival patterns of the cohort based on WHO clinical stage categories. The overall mortality rate provides an incidence density of 2.03 deaths per 100 person years (95% CI 1.64–2.50). Out of a total of 86 deaths over 60 month period; 63 (73.3%) died during the first 12 months, 10 (11.6%) during the second year, and 10 (11.6%) in the third year of follow up. When the follow-up period was divided into 1-year time bands (from ART initiation through 1 year, from 1 year through 2 years, and from 2 years though 3 years), the mortality rates per 100 person years at risk were 55.67 (95% CI 43.48–71.26), 4.03 (95% CI 2.17–7.51), and 0.31 (95% CI 0.18–0.54) respectively.

### Predictors of mortality

In univarite analysis, factors associated with the predictor of mortality were previous weight loss of more than 10%, bedridden functional status at baseline, less than 200 CD4 cells/ml and WHO stage IV patients. Those patients who reported to have lost a weight of more than 10% at baseline were 5 times more likely to die compared to those who did not (hazard ratio, HR 4.41; 95% CI 1.08–17.92). Those patients who were bedridden or non-ambulatory at the initiation of treatment were five times more likely to die compared with those that were working (HR 5.33; 95% CI 3.05–9.30). Those patients whose CD4 cell count lied between 201–300 were 58% less likely to die as compared to those patients whose CD4 count was less than 200 cells per milliliter (HR 0.42; 95% CI 0.19–0.92).

In the multivariate analysis, five baseline factors were independent predictors of mortality. WHO stage IV patients were 3 times more likely to die compared to stage I and II patients (HR 3.19; 95% CI 1.51–6.76). Bedridden patients were four times more likely to die compared with those patients who are working (HR 4.09; 95% CI 2.12–7.90). Patients who reported to have lost more than 10% of their weight at baseline were 5 times more likely to die compared to those patients who did not (HR 4.93; 95% CI 1.20–20.41). Patients whose CD4 cell counts between 201–300 were 60% less likely to die compared to those whose CD4 counts less than 200 (HR 0.40; 95% CI 0.17–0.93). Those patients with primary education were almost 3 times more likely to die than illiterate counterpart (HR 2.79; 95% CI 1.26–6.16) (Table 2).

**Table 2:** Predictors of mortality among a sample of HIV infected cohorts on anti-retroviral treatment in eastern Ethiopia

Independent variables	Unadjusted HR (95% CI)	P-value	^¥^Adjusted HR (95% CI)	P-value
Age	1.02 (0.99–1.04)	0.131	1.02 (0.99–1.05)	0.156
Sex				
Male	1.00		1.00	
Female	1.05 (0.68–1.64)	0.819	1.16 (0.68–2.00)	0.584
Religion				
Muslim	1.00		1.00	
Christian	0.85 (0.51–1.42)	0.539	0.98 (0.55–1.75)	0.939
Employment				
Employed	1.00		1.00	
Not employed	1.05 (0.66–1.67)	0.827	0.94 (0.58–1.52)	0.788
Marital status				
Not married	1.00		1.00	
Married	0.92 (0.60–1.41)	0.694	0.97 (0.60–1.56)	0.884
Education				
No education	1.00		1.00	
Primary	1.58 (0.79–3.16)	0.193	2.79 (1.26–6.16)	0.011
Secondary or above	1.42 (0.70–2.87)	0.331	1.40 (0.61–3.22)	0.435
Baseline functional status				
Working	1.00		1.00	
Ambulatory	1.28 (0.77–2.13)	0.337	1.14 (0.64–2.04)	0.651
Bedridden	5.33 (3.05–9.30)	0.000	4.09 (2.12–7.90)	0.000
10% weight loss				
No	1.00		1.00	
Yes	4.41 (1.08–17.92)	0.038	4.93 (1.20–20.41)	0.027
CD4 category				
≤ 200	1.00		1.00	
201–300	0.42 (0.19–0.92)	0.029	0.40 (0.17–0.93)	0.034
>300	0.67 (0.24–1.82)	0.428	0.86 (0.30–2.41)	0.77
WHO category				
Stage I and II	1.00		1.00	
Stage III	1.35 (0.77–2.36)	0.294	1.60 (0.85–3.04)	0.15
Stage IV	3.35 (1.80–6.24)	0.000	3.19 (1.51–6.76)	0.002

^¥^Global test of proportionality of hazards derived from Schoenfeld residuals not significant (df = 13, ch2 = 13, p = 0.108).

## Discussions

The findings indicate that from the registered cohort, there were 86 deaths in 4234.8 years of retrospective follow up, providing an incidence density of 2.03 deaths per 100 person years (95% CI 1.64–2.51). About 210 (14.0%) patients were lost to follow up. Factors that were associated with mortality were 10% weight loss, bedridden functional status at baseline, ≤200 CD4 cells/ml and advanced stage patients.

Long-term retention of patients in antiretroviral treatment is a prerequisite for achieving any adherence at all. Various studies have shown that mortality during the first 6 months after initiating ART is much higher than in developed countries and retention of patients in programs is poor [4,13]. However, most longitudinal studies conducted in Africa have been either short-term or have involved small numbers of participants.

In our study, the overall mortality rate of 2.03 deaths per 100 person years is lower or comparable to that reported elsewhere; however the mortality rate at the first year (55.67; 95% CI 43.5–71.3; or 4.3%) is high. In the ART-LINC Collaboration, which analyzed data from 18 cohorts across the developing world, mortality averaged 4.2% across all 18 cohorts in the first year after initiation [11]. In Yaounde, Cameroon, from 312 patients, the incidence rate of mortality rate was 21.2 per 100 person years (95% CI 15–31) [14]. In southern Ethiopia, the mortality rate was 15.4 per 100 person-years of observation [12]. The majority or 74.1% of the deaths occurred in the first year after treatment. The study highlights the high early mortality in ART cohorts in resource-limited settings that has been observed by other groups in similar settings [4,15,16].

The primary causes of death in AIDS patients could be the causes such immune reconstitution syndrome and opportunistic infections as a result of very weak immunity levels. In our study one of the factors associated with early mortality is late presentation for ART. This may also account for the high rate of death in the first year. According to reports, patients that start ART at WHO Stage III and IV are at an increased risk of dying [17,18]. Furthermore, early mortality risk is higher among those with low CD4 cell count [4,19]. The CD4 count is a proxy indicator of severity of disease which corresponds to the functional status and reflects the immune state of patients [20]. In our study, the functional status of patients at the entry level had a positive correlation with their disease stage and negative correlation with CD4 count. In a report from Hong Kong, there was a 79% reduction in mortality among ART taking patients with CD4 counts of less than 200/ml [21]. The majority of morbidity and mortality seen among individuals starting ART with low baseline CD4 cell count occurs during the first 3–6 months on treatment [2,3,15,16,22]. In one study patients initiating ART with a base-line CD4 cell count of less than 50 cells/mm3 had a 3.2-fold higher mortality rate (p 0.004) compared with patients with a CD4 cell count between 51 and 200 cells/mm3 at the time of ART initiation [23]. Patients starting treatment with CD4 cell count below 100 cells/mm3 were at significantly greater risk of death during the follow-up period (OR 2.69; 95% CI 1.12–6.44) [12].

Nutritional and physical status can predict early mortality. In our study, 10% weight loss determines mortality during the course of treatment. In a district hospital in Ethiopia, weight loss was seen in about a third of patients who survived up to the fourth week, and it was associated with increased death [18]. Higher body weight at baseline was found to be associated with lower risk of mortality with a HR of 0.58 for weight groups of 40–50 kilograms compared with the reference of less than 40 kilograms, and HR of 0.25 for groups ≥60 kilograms compared with the reference groups (p < 0.013). The two year patient survival was significantly related with co-trimoxazole initiation at or before treatment, clinical stage IV disease, working functional status and weight greater than 60 kilograms [24]. In another study, baseline body mass index (BMI) of less than 18.5 was independently associated with early mortality risk [19].

About 61% of patients in this study had education below secondary school. According to reports, low educational level among patients is a contributing factor to late presentation for ART [25]. This is understandable since the more educated a patient is the better their understanding of the disease state and comprehension of instructions given on drug usage. These could enhance treatment outcomes [26]. A report from British Columbia reported a protective effect of educational level on mortality from all causes among intravenous drug users receiving anti-retroviral treatment [27,28]. Most reports suggest that low educational level has consistently been associated with higher mortality, both overall and cause-specific [29,30]. In our study lower educational level was associated with a higher risk of mortality. However, patients with primary education seem to be at higher risk of death compared to those with no education. This is an interesting finding for which we could not find explanation due to the nature of our data, however it needs further investigation in future research.

This study has limitations. CD4 cell counts and HIV RNA measurements were not available for all patients because of cost issues. Diagnostic tests that would confirm the presence of certain opportunistic infections were limited; as a result we were not able to include these in our analysis. The retrospective cohort study design limited our ability to gather data about factors that may influence the risk of mortality, for instance factors such as lack of social supports networks, disclosure of infection status and depression. Data was collected from those who were attending ART centers mostly through self-reporting and hence may have reporting and recall bias. In ART programs of developing countries like Ethiopia, poor ascertainment of deaths and recording of information on losses to follow up may lead to underestimation of mortality rates.

In conclusion, we detected a relatively lower level of mortality among the cohort of patients on antiretroviral treatment in eastern Ethiopia. Previous history of weight loss, bedridden functional status at baseline, low CD4 cell count and advanced WHO patients had a higher risk of death among the retrospective cohort. So early initiation of ART while CD4 counts are higher and opportunistic infections limited, provision of nutritional support and strengthening the food by prescription initiative, and counseling of patients for early presentation during testing for HIV is recommended.

## Competing interests

All authors declare that they have no competing interest associated with the publication of this manuscript.

## Authors’ contributions

AAR and SB contributed significantly in the design, manuscript writing and review. AAR conceived and designed the study, collected data in the field, performed the data analysis, interpreted the data, and drafted the manuscript and critically reviewed it. TD was significantly involved in data collection and has reviewed the manuscript. All authors approved and read the final manuscript.
